# Taking knowledge for health the extra mile: participatory evaluation of a mobile phone intervention for community health workers in Malawi

**DOI:** 10.9745/GHSP-D-13-00141

**Published:** 2014-02-06

**Authors:** Natalie Campbell, Eva Schiffer, Ann Buxbaum, Elizabeth McLean, Cary Perry, Tara M Sullivan

**Affiliations:** a Management Sciences for Health, Arlington, VA, USA; b World Bank, Washington, DC, USA; c Public Health Consultant, Boston, MA, USA; d Management Sciences for Health, Boston, MA, USA; e Johns Hopkins Bloomberg School of Public Health, Center for Communication Programs, Baltimore, MD, USA

## Abstract

In Malawi, where the majority of the population resides in rural areas, community health workers (CHWs) are the first, and often only, providers of health services. An assessment of health information needs, however, found that these frontline workers often lacked essential health information. A pilot project, implemented in 2 rural districts of Malawi between 2010 and 2011, introduced a mobile phone system to strengthen knowledge exchange within networks of CHWs and district staff. To evaluate the mobile phone intervention, a participatory evaluation method called Net-Map was used, an approach built on traditional social network analysis. Together, CHWs and district personnel discussed information needs and gaps and the roles of different actors in their information networks. They then used drawings and 3-dimensional objects to create baseline and endline maps showing the linkages and levels of influence among members of the information network. Net-Map provided them with powerful evidence of differences before and after the mobile phone initiative. At baseline, CHWs were not mentioned as actors in the information network, while at endline they were seen to have significant connections with colleagues, beneficiaries, supervisors, and district health facilities, as both recipients and providers of information. Focus groups with CHWs complemented the Net-Map findings with reports of increased self-confidence and greater trust by their communities. These qualitative results were bolstered by surveys that showed decreases in stockouts of essential medicines, lower communication costs, wider service coverage, and more efficient referrals. As an innovative, participatory form of social network analysis, Net-Map yielded important visual, quantitative, and qualitative information at reasonable cost.

## Abstract (precis)

A participatory evaluation process called Net-Map showed that providing community health workers (CHWs) with mobile phones and essential technical information changed CHWs, from passive recipients of information with little influence to active information agents who sought and provided information to improve health services.

## BACKGROUND

Malawi, one of the world's poorest countries, has health indicators that are consistent with a weak economy, particularly in the 2 health areas addressed by the Knowledge for Health (K4Health) Malawi project—family planning/reproductive health and HIV/AIDS. Four examples illustrate the situation:

- Malawi's total fertility rate is 5.7.^1^
- Despite considerable progress in reducing maternal mortality over the past few years, 460 women still die from pregnancy-related causes per 100,000 live births.^1^^,^^2^
- In 2011, Malawi's HIV prevalence rate was 10%,^3^ the ninth highest in the world.^4^
- Between 2007–2012, the contraceptive prevalence rate was 46%.^5^

Of the 16 million people in Malawi, 85% reside in rural areas, where health conditions are generally worse and where vital family planning/reproductive health and HIV/AIDS services are not readily accessible. The HIV epidemic has taken a particularly large toll in rural communities that have limited access to prevention and treatment and that are often bearing the brunt of caring for people living with HIV/AIDS who move back to their villages from an urban residence.

Malawi has approximately 2 doctors per 100,000 population,^6^ far fewer than neighboring African countries such as Zambia, with 10 doctors per 100,000, Botswana with 30, and Namibia with 40. The ratio of nurses to population in Malawi is equally low: 37 per 100,000 compared with 70 per 100,000 in Zambia and 280 in Botswana and Namibia.^7^ In rural areas, community health workers (CHWs) are the first—and often only—providers of health services.

In Malawi, community health workers are the first, and often only, providers of health services.

Interventions by CHWs have repeatedly demonstrated the potential to reduce mortality and morbidity in sub-Saharan Africa. The World Health Organization (WHO) and the Millennium Villages Project have called for the rapid scale up of CHWs in sub-Saharan Africa.^8^^,^^9^ In March 2013, a Technical Taskforce of the Earth Institute at Columbia University released a report calling for the training and deployment of 1 million CHWs in sub-Saharan Africa by 2015.^10^ These recommendations are particularly appropriate in Malawi, given the shortage of higher-level health professionals and the acute health needs of people living in rural areas.

Malawi has a countrywide system of CHWs, classified as either community-based distribution agents (CBDAs) or health surveillance assistants (HSAs). CBDAs are volunteers selected by their communities to provide family planning counseling, oral contraceptives, and condoms. HSAs are salaried community-based health workers who deliver immunizations, family planning (including injectables), well-child visits, and disease surveillance. They play a critical role in linking residents of remote villages with formal health services located a considerable distance away.

An assessment of health information needs conducted early in the K4Health Malawi project showed that in Nkhotakota and Salima Districts, these frontline workers often lacked essential up-to-date knowledge about the health areas for which they were responsible.^11^ Although there was frequent communication and knowledge exchange between national and district levels, this did not hold true between districts and communities. There was no coherent system or central location where CHWs could find complete and current information about family planning/reproductive health and HIV/AIDS.

Community health workers often lack essential health information needed to do their work.

Mobile phone towers exist throughout Malawi, and some service providers own mobile phones and use them for both short message service (SMS) text messages and voice calls.^11^ Although very few CHWs in Nkhotakota and Salima Districts personally owned and used mobile phones at the time of the needs assessment, project staff agreed that the large and growing use of the SMS technology offered great potential as a channel for knowledge exchange.

Other projects in sub-Saharan Africa have been successful with using mobile phones to advance health goals. Among the successful applications most relevant to the K4Health Malawi project were those to promote adherence to antiretroviral treatment,^12^^,^^13^ to ensure compliance with malaria treatment guidelines,^14^ and to facilitate surveillance and data collection.^15^ A pilot study of mobile phone use for treatment of tuberculosis in Malawi found savings in workers' time and money and a doubling of treatment capacity.^16^ Furthermore, a review article on the use of mobile phone messaging in South Africa concluded that mobile phones are a practical, low-cost tool to deliver information that supports high-quality health care.^17^

This article briefly describes the mobile health intervention of the K4Health Malawi project and explores the effects of the intervention on knowledge exchange, focusing particularly on the qualitative and quantitative data collected through a participatory action research methodology called Net-Map. The article also explores some of the impacts that the Net-Map sessions had as interventions in and of themselves.

## PROGRAM DESCRIPTION

Between June and October 2010, the project trained and provided mobile phones, solar chargers, and airtime to 253 CHWs in Nkhotakota and Salima Districts—30% of all CHWs in the 2 districts combined. An additional 385 CHWs received phones, chargers, and training during a second distribution in November 2010, bringing SMS coverage to 77% of health workers in both districts, targeting those whose homes were farthest away from health centers. About two-thirds of the participating CHWs were HSAs, and about one-third were CBDAs.

**FIGURE 1.**
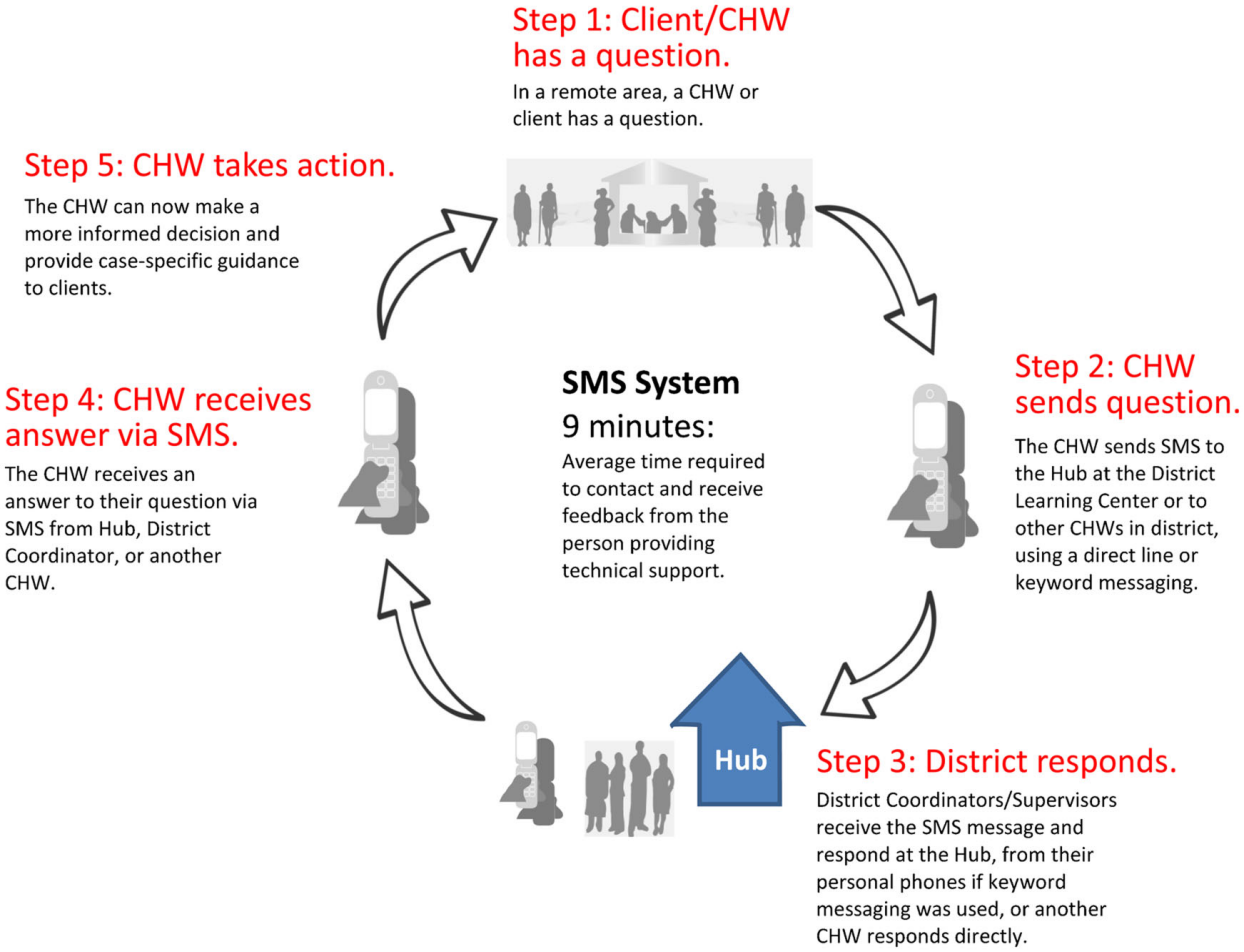
SMS Network in Nkhotakota and Salima Districts of Malawi Abbreviations: CHW, community health worker; SMS, short message service.

The mobile phones linked CHWs with each other and with district supervisors/coordinators through an SMS hub located at the 2 District Hospitals (Figure 1).

In addition to providing new channels for communication (mobile phones and the SMS Hub), the system also filled these channels with essential technical information. The communication flow encompassed requests from health workers as well as prompt replies from district supervisors and coordinators. When clients approached health workers with urgent questions, or when workers needed to restock contraceptives, the workers could use their mobile phones to send a text message to the Hub, where a district coordinator or supervisor would be assigned to read and respond to messages. Alternately, a worker could reach a specific supervisor directly by using defined keywords, which the Hub would recognize and forward to the phone of the supervisor. The CHW could also use the system to contact a fellow worker within the network to ask a question or make a request.

One of the reasons that the project chose this mHealth intervention was because of its low cost. The mobile phones were US$27 each, and the solar chargers were US$7 each. Compared with other projects using more sophisticated and expensive phones, these phones allowed project costs to remain low.

## METHODS

To assess outcomes of the K4Health Malawi project, we implemented a comprehensive evaluation plan that used several qualitative and quantitative methodologies, including Net-Map research, Lot Quality Assurance Sampling (LQAS), and focus group discussions. This article provides an in-depth description of the Net-Map methodology because of its innovative nature and of the rich findings that it produced.

### What Is Net-Map?

Net-Map is a process adapted from social network analysis, a key technique used in modern sociology. The basic idea of social network analysis is that people do not act alone; their actions and the impact of those actions are determined by the social network structure in which they are embedded. This structure is viewed as a group of related actors, represented as nodes, and their interactions, represented as links between those nodes.^18^

Net-Map helps to identify the social network structure in which certain stakeholders operate as well as levels of influence.

Net-Map starts with a simple pen and paper-based form of social network mapping, designed to highlight formal and informal interactions among key actors in a network.^19^ Net-Map extends the traditional scope of social network analysis by including power or influence mapping, a method by which actors in a network discuss and analyze the different kinds of influence that exist within their network, reach agreement on the extent to which each actor in the network exerts his/her influence, and use a simple visual tool to show what they have found.^20^ The definition of influence in a Net-Map context is based on Max Weber's definition of power—that is, the ability of an actor to achieve one's goal in a social setting, irrespective of the means used.^21^

Because participants draw network maps together, Net-Map promotes knowledge exchange among varied actors or stakeholders. They have to discuss, debate, and come to agreement before they draw the connections. Their discussion provides rich data about the way the network functions. The process helps participants to understand, visualize, monitor, evaluate, and improve situations in which many different actors influence outcomes.

Net-Map is both a research and knowledge exchange tool, because participants discuss and draw the network maps together.

In this way, Net-Map combines visual results (network maps), quantitative results (network data), and qualitative results (network narratives). It creates a setting in which network members are participants in, rather than objects of, research.

### Net-Map Participants

We conducted Net-Map through half-day workshops held in Nkhotakota and Salima at the beginning of the project in May 2010 and at endline in June 2011. In 2010, the workshops in each district supplemented the K4Health Malawi needs assessment and yielded baseline information for the mobile phone intervention; participants provided rapid appraisals, or snapshots, of the flow of technical health information to and from CHWs within their networks and assessed bottlenecks, strengths, and opportunities in information flows. In the 2 endline workshops in 2011, participants reviewed these factors and tracked how they had changed since the beginning of the mobile phone intervention.

Participants were selected based on their membership in key stakeholder groups related to the technical focus of their work in HIV/AIDS and family planning/reproductive health in Malawi, including both types of CHWs (HSAs and CBDAs) as well as representatives of district health offices and health facilities. The same stakeholder groups were represented in both the 2010 and 2011 workshops, although in some instances different individuals represented the stakeholder group. A limit of 15 participants per workshop was necessary to ensure substantive discussions and output.

### Net-Map Procedures

The Net-Map process consisted of 4 steps in both the 2010 and 2011 workshops (Table). In 2011, one additional question in Step 4 and one extra step was added to allow for comparisons from baseline to endline.

**TABLE.** Net-Map Interview Structure

**Steps**	**Questions**
Overall Question	Who plays a role in improving flows of technical information on HIV/AIDS and reproductive health to health care service providers?

Step 1	**Name generator**: List the actors involved.

Step 2	**Link generator**: What are the critical information flows for Malawi in improving health care for HIV/AIDS and reproductive health? Specific links:
	• Who provides technical information on HIV testing?
	• Who provides technical information on family planning?

Step 3	How influential is each actor in improving these information flows?

Step 4	**Discussion questions**
	**In 2010:**
	a. Who are the critical actors?
	b. What are the critical linkages?
	c. How can we ensure improved information flows?
	**In 2011:** a, b, and c, with an added question:
	Of the links, which have been strengthened due to the mobile phones?

Step 5	**Comparison of maps between 2010 and 2011**
	• What are some of the significant changes you see?
	• What do you think are the reasons for these changes?

**Step 1: Identifying the actors in the network.** Participants identified all actors in their networks who were involved in the production, exchange, and storage of information on family planning/reproductive health and HIV/AIDS on labeled notes and placed the notes on a large sheet of paper.

**Step 2: Linking the actors.** The participants described the connections between the actors in terms of the flow of technical information that CHWs send or receive in the context of their jobs. The facilitator drew arrows to depict these connections, using different colored arrows for family planning/reproductive health and HIV/AIDS information. Drawing these links help identify providers, conduits, and recipients of knowledge. If participants disagreed about whether to draw a link, the facilitator encouraged them to discuss, explain, and reach agreement in the group. The maps that they drew were the result of this deliberative process.

**Step 3: Mapping the influence.** In this step, participants used checker pieces to create “influence towers” next to the label representing each actor. The facilitator clarified that “influence” in this sense did not just represent formal decision-making authority but also more informal ways of influencing, such as giving advice or incentives or bending the rules. The question was: “How strongly does this actor influence knowledge flow on family planning/reproductive health and HIV/AIDS in the Malawi health system?” This reflects the insight that one major problem in the Malawi health system is the asymmetry of information and lack of knowledge flow between the most and least knowledgeable parts of the system. The participants ranked each actor with an influence value from 0 to 10—the greater the influence, the higher the tower. Through facilitated in-depth discussion, workshop members came to agreement about the perceived influence level of each actor.

**Figure:**
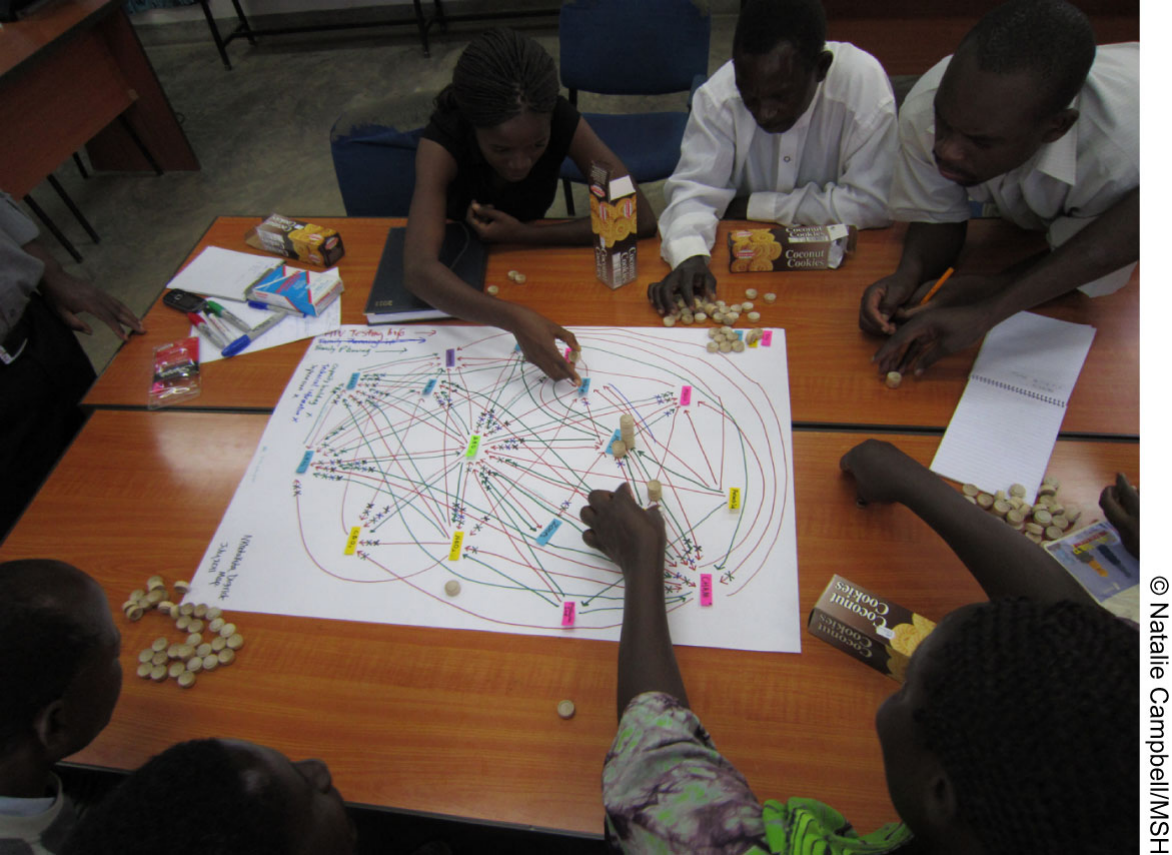
Net-Map participants in Nkhotakota, Malawi, produce a 3-dimensional drawing of their knowledge exchange network.

**Step 4: Facilitating the discussion.** The facilitator led a discussion focused on the 3-dimensional depiction of the network just created: the actors, their links, and their level of influence. Using this visual tool as a reference, the facilitator asked about the “how” and “why” of the network as it evolved in front of participants' eyes.

**Step 5: Comparing the maps.** At the 2011 workshop, participants compared the map they drew with the map of their network from 2010. The facilitator again guided the discussion of changes between the maps and possible reasons for these changes. In addition to asking about the “how” and “why” of the network, the participants focused on a new question: “Of the links, which have been strengthened as a channel for information and knowledge flow due to the mobile phones?”

### Analysis of Net-Map Data

**FIGURE 2.**
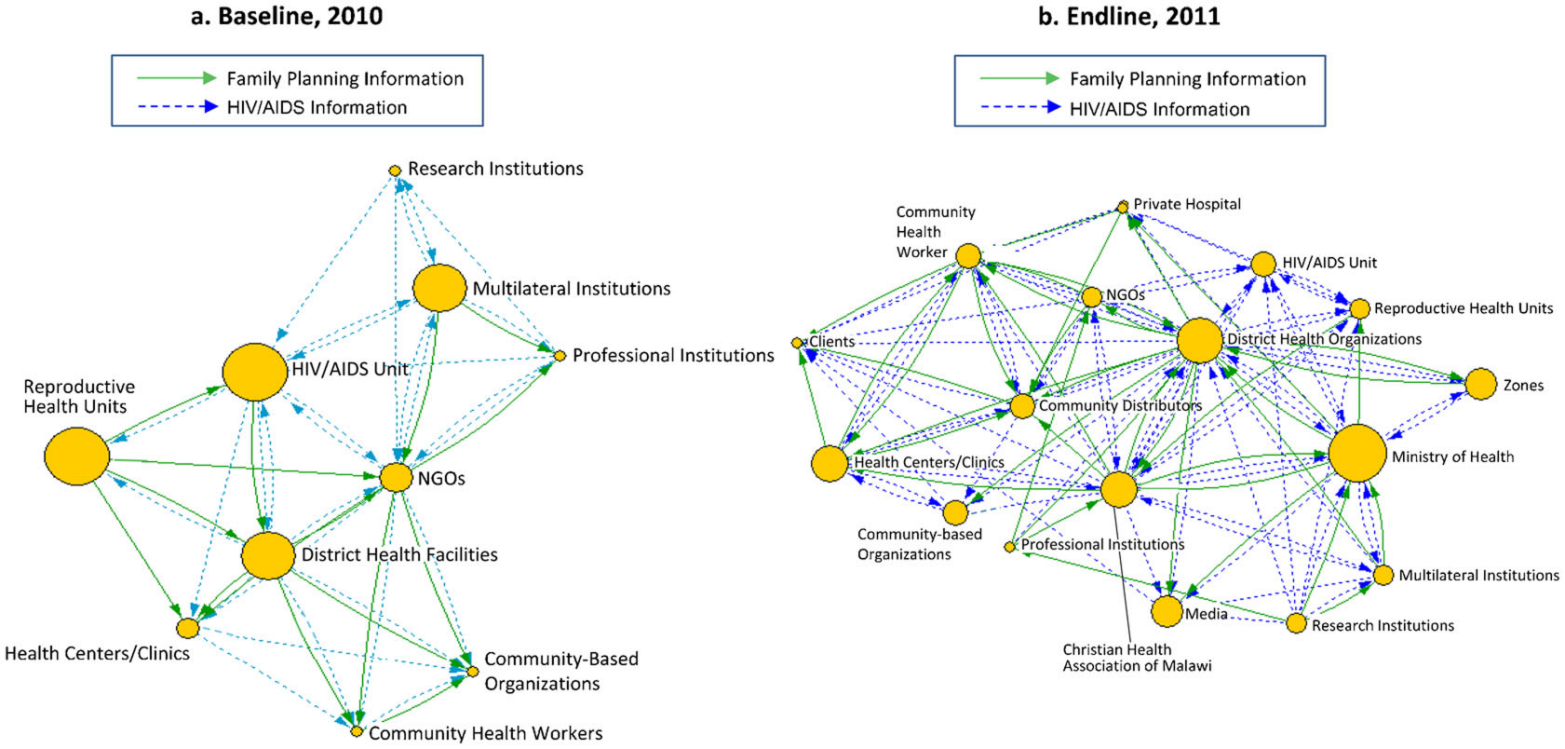
Baseline and Endline Net-Maps of Information Flows With District-Level Stakeholders in Nkhotakota, Malawi

**FIGURE 3.**
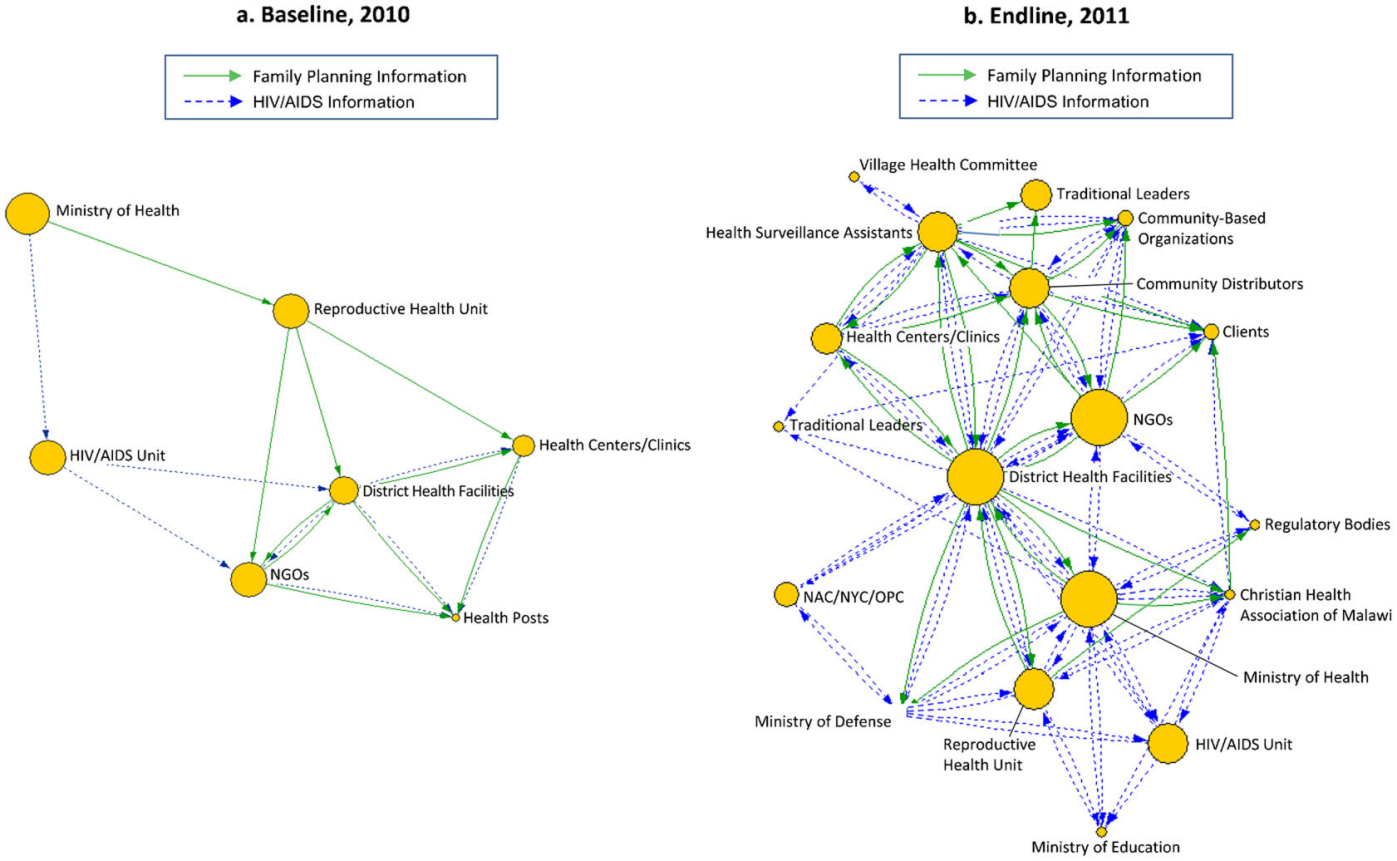
2010 Baseline and Endline Net-Map of Information Flows With District-Level Stakeholders in Salima, Malawi

After each workshop, project evaluators entered the data from the hand-drawn maps into VisuaLyzer™ software to create computer-generated maps. The size of each node on the maps corresponded to the height of the influence tower for that actor, as seen in the baseline and endline maps for Nkhotakota (Figure 2) and Salima (Figure 3).

The evaluators then used the data on the maps to calculate degree centrality for Nkhotakota and Salima in 2010 and 2011. Degree centrality is a quantitative measure that is basic to social network analysis. It defines the positions of the actors in a network by a score that indicates the number of links each actor has with other network members. In this way, it is a measure of connectedness: the extent to which the actor can access needed information from and provide relevant information to other network members.

In the Nkhotakota and Salima degree centrality graphs, scores are expressed in the percentages along the y axis. A score of 100%, for example, would indicate that an actor was linked to 100% of the network members and was, thereby, a critical recipient and provider of information; a very low score would indicate few network linkages, little access to essential information, and minimal or nonexistent involvement in the flow of information.

As the graphs demonstrate, the measure of degree centrality can be split between in-degree (how many links are directed to each actor) and out-degree (how many links each actor directs to others). In both Nkhotakota and Salima, the Net-Map workshop discussions confirmed that having a high out-degree (giving information to many actors) was an indication of higher status, while having a high in-degree (being mainly the recipient of information) was typical for lower-status actors.

### Other Evaluation Methods

In addition to the Net-Map research, the project conducted LQAS surveys with CHWs in the 2 intervention districts as well as in a control district with a similar socioeconomic profile but without access to SMS technology. These surveys were carried out before expansion of the SMS network (November 2010) and at endline (May–June 2011).

For the pre-expansion LQAS, each of the 3 districts was divided into 5 supervision areas. By endline, the project understood how the districts actually worked through the SMS activities, and the 2 intervention districts and control district were divided into 4 supervision areas.

For both LQAS surveys, respondents were sampled from each supervision area using a random number table and Ministry of Health lists of all HSAs and CBDAs. For the pre-expansion survey, this yielded a total sample size of 285, of which 65% were HSAs and 35% were CBDAs. At endline, it yielded a total sample size of 228, of which 63% were HSAs and 37% were CBDAs.

To supplement the quantitative data, we also conducted focus group discussions, before and after the intervention, with CHWs who had been given mobile phones but had not participated in the Net-Map workshops. In these discussions, the participants described how often, for how long, for what purpose, and at what cost they communicated with each other and with their district-level supervisors before and after having mobile phones.

## RESULTS

### Effects on Knowledge Exchange

**FIGURE 4.**
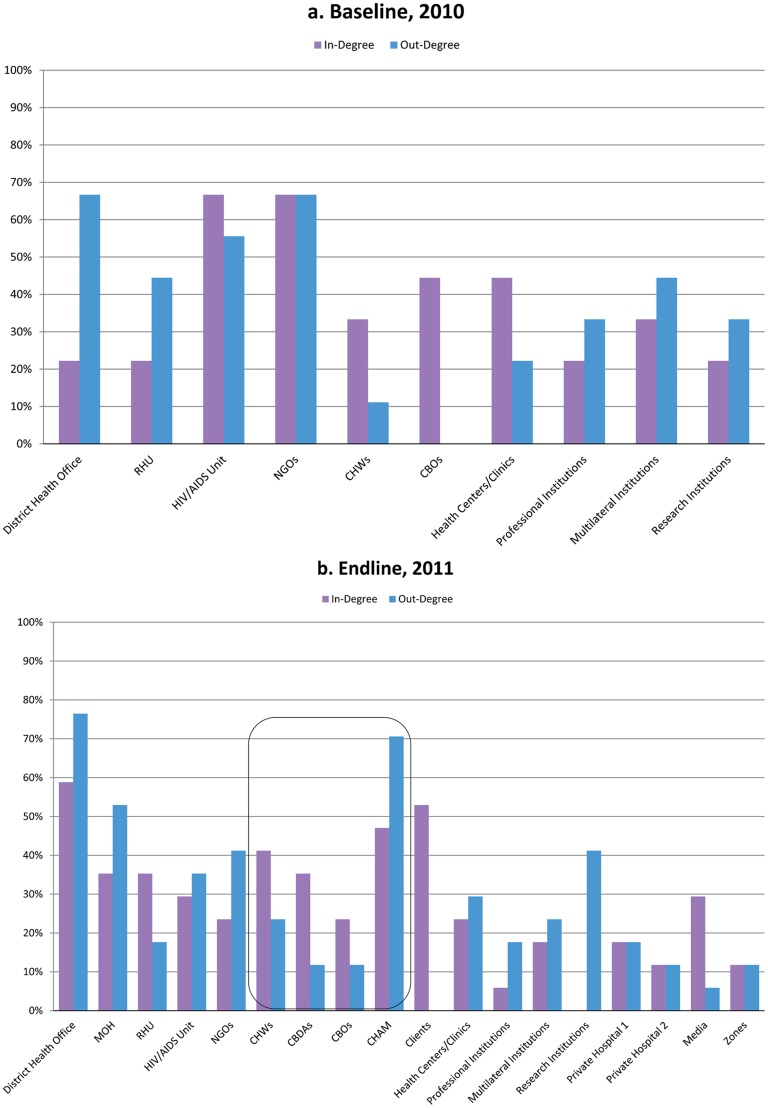
Baseline and Endline In-Degree and Out-Degree Centrality Values for Nkhotakota, Malawi Abbreviations: CBDAs, community-based distribution agents; CBOs, community-based organizations; CHAM, Christian Health Association of Malawi; CHWs, community health workers; MOH, Ministry of Health; NGOs, nongovernmental organizations; RHU, Reproductive Health Unit.

**FIGURE 5.**
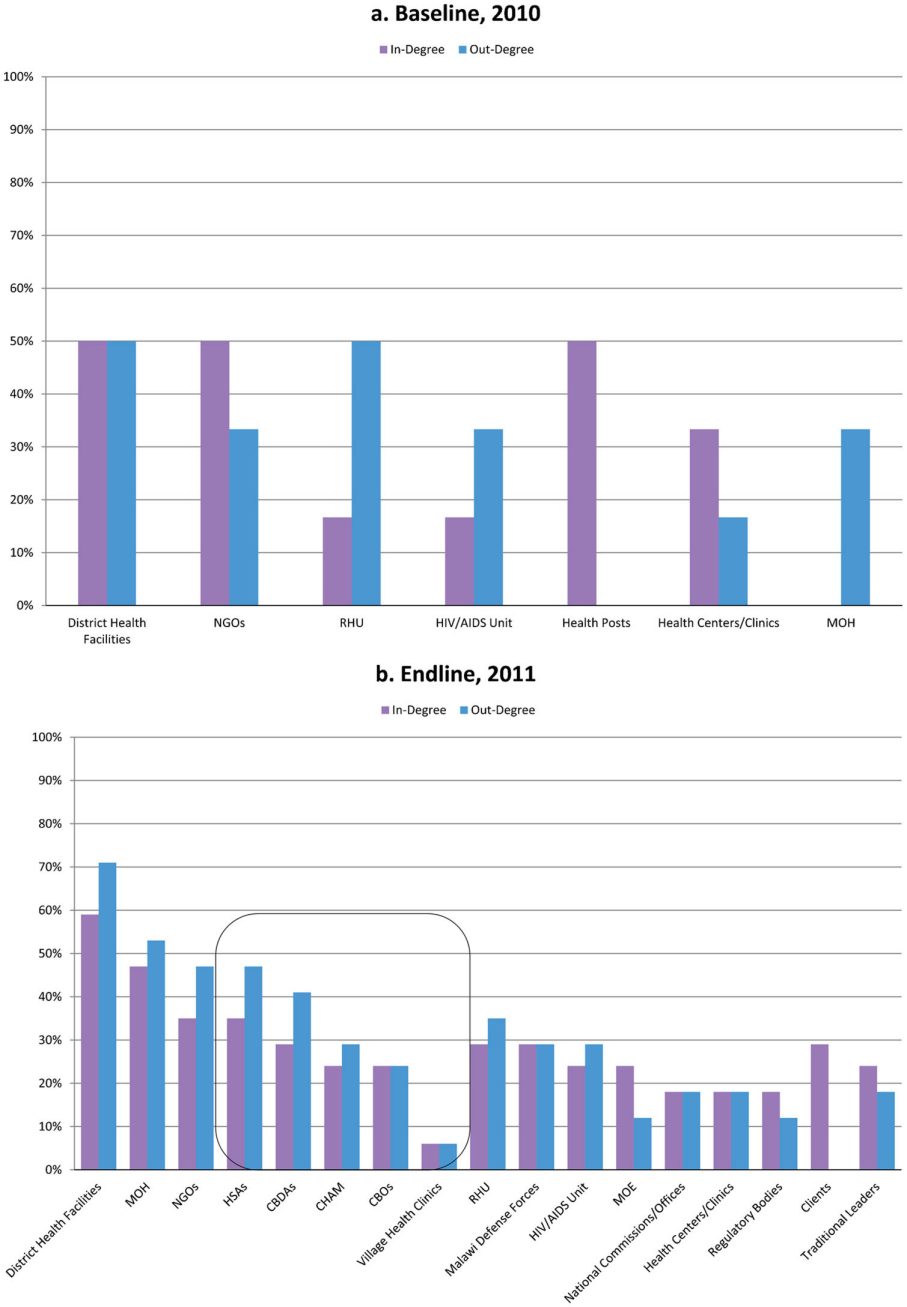
Baseline and Endline In-Degree and Out-Degree Centrality Values for Salima, Malawi Abbreviations: CBDAs, community-based distribution agents; CBOs, community-based organizations; CHAM, Christian Health Association of Malawi; HSAs, health surveillance assistants; MOE, Ministry of Education; MOH, Ministry of Health; NGOs, nongovernmental organizations; RHU, Reproductive Health Unit.

The Net-Map process clearly demonstrated the effects of the mobile phone intervention, which was reinforced by the supporting quantitative results from the LQAS methodology. Both baseline district network maps in 2010 (Figure 2a and Figure 3a) revealed that knowledge exchange was extremely hierarchical; high-level actors had high influence, and information flowed from top to bottom. The maps and degree centrality values showed minimal communication, primarily in-degree at the district level; the only exception was for HIV/AIDS information going to community-based organizations, which were equally remote from the actors of greatest influence. In addition, the baseline maps were very sparse, with few actors and few linkages between actors, indicating weak information flows around family planning/reproductive health and HIV/AIDS. The CHWs were particularly affected by this situation: they were barely represented by a small node in Nkhotakota and were completely absent in Salima. The small size of their nodes (indicating low influence) and the largely in-degree centrality (Figure 4a and Figure 5a) showed that before the mobile phone intervention, CHWs had virtually no influence and were largely recipients of information from very few sources.

Before the mobile phone intervention, community health workers had virtually no influence in the knowledge exchange network.

The contrast with the complex flow of information seen in the 2011 endline maps (Figure 2b and Figure 3b) and degree centrality graphs (Figure 4b and Figure 5b) was striking. In both districts, the nodes for CHWs on the maps were considerably larger, and the degree centrality graphs identified different types of CHWs at varied work sites where mobile phones had been distributed (outlined with a box in Figure 4b and Figure 5b). These CHWs—who had not shown up at all in the centrality graphs a year earlier—now had considerable presence and a balance of in- and out-degree centrality. CHWs who were using mobile phones had turned from passive recipients of information to active information agents who sought and provided information back up the hierarchy and across the network to one another as colleagues on the front lines. The arrows on the 2011 maps (Figure 2b and Figure 3b) showed information from most actors feeding into the nodes for CBDAs and HSAs, and similarly many arrows feeding back out from these agents to district health facilities and NGOs as well as to traditional leaders and clients.

After the mobile phone intervention, community health workers had turned into active information agents.

The centrality graph for Salima in Figure 5b shows that in 2011, CHWs were not only included in the network but were differentiated as either HSAs or CBDAs. Their out-degree centrality was higher than their in-degree, reflecting their new status as sources of important information for each other and for their supervisors, most often regarding the health status of a community member, the vital statistics of a sick child, or low stocks of contraceptives.

On the 2011 map for Nkhotakota (Figure 4b), several new actors appear, and the arrows depicting knowledge exchange are far more extensive. Participants in that Net-Map workshop added CBDAs to their map as a separate entity within the broad category of CHWs. They also added the Christian Health Association of Malawi (CHAM) and private hospitals to the map, a reflection of the fact that HSAs stationed in private and CHAM hospitals were among the health workers equipped with mobile phones—thus giving them a new voice and role within the network.

In workshop and focus group discussions, participants pointed out that the use of mobile phones gave them the means to communicate not only with supervisors at the district hospital, the CHAM hospital, and private hospitals but also with their colleagues. CHWs could now get immediate help for their clients by sending a message to the Hub and getting a rapid response or by sending questions to district hospital staff and receiving important technical information with little delay.

Participants in workshop and focus group discussions in 2011 described the increased influence of CBDAs and HSAs in the past year. They explained that all actors in the district were now relying on these workers to provide clients with HIV/AIDS and reproductive health information.

### Effects on Health Services

The Net-Map workshops and focus group discussions also revealed the direct effect of the mobile phone intervention on health services. The CHWs cited their ability to get immediate help for their clients by sending a message to the Hub and getting a rapid response. CHWs explained that timely responses from district hospital staff to CHWs' requests for important technical information resulted in gains in expertise. They also described the reduction in stockouts that resulted from expediting the timely reporting of family planning/reproductive health and HIV/AIDS commodity shortages.

In addition to improving knowledge exchange, the mobile phone intervention also had direct effects on use of health services.

Equally important were the less tangible results that the CHWs reported: increased self-confidence and increased trust between them and their communities. There was an increase in prompt responses to emergencies (obstetric) and outbreaks (measles) and to queries from CHWs to their supervisors. CHWs reported a wider service coverage accompanied by lower costs. The LQAS study also confirmed a 90% decrease in travel costs because health workers could text, instead of renting a bike, to obtain technical support. Additionally, the intervention reduced the time spent getting information from 1.2 days to 9 minutes, with cost savings from, on average, 200 Malawian kwachas to 10 kwachas to get technical support.

### Limitations

When drawing the information flow networks during Net-Map sessions, participants lumped together a number of different kinds of “information flow,” from information about stockouts to information concerning treatment methods. Finer differentiation between different kinds of information and a discussion about the quality of information exchanged would have deepened the insights from the workshops. Ideally, a control group would have been included to strengthen the case for causal attribution.

With the LQAS, we could not compare pre-expansion and endline supervision areas individually due to the change in methodology in identifying supervision areas between the 2 time periods. However, we were able to aggregate data across all the supervision areas and compare indicators. The project also used endline data to determine the degree to which individual supervision areas in the 2 intervention districts met coverage benchmarks for key project indicators.

## CONCLUSIONS

The ability of the K4Health Malawi project to provide 77% of the CHWs in both intervention districts with mobile phones and to successfully train them in using the phones within 1 year is a remarkable outcome in such a short time frame. The most important shift that occurred due to the mobile phone intervention was that instead of being only occasional recipients of information from the district level, CHWs became initiators of information flows through their stockout messages and clinical questions. This is perhaps the greatest legacy of this project in a predominantly rural country where CHWs serve as a crucial link with clients.

These results were achieved through a 2-pronged approach: providing new channels for communication (mobile phones and SMS Hub) and supplying these channels with content and processes for knowledge sharing (requests from health workers and prompt replies from the district supervisors and coordinators). The Net-Map workshop discussions confirmed that neither of these 2 interventions alone—merely giving out mobile phones or compiling information without developing channels for knowledge exchange—would have been as effective. Future projects aiming to improve knowledge flows through information technologies should focus both on developing technically feasible and robust channels and on encouraging knowledge sharing behavior.

Future knowledge exchange projects should focus on developing appropriate communication channels as well as on encouraging knowledge sharing behavior.

As a tool for action research, the Net-Map process yielded visual, quantitative, and qualitative evaluation data, and it also enhanced the sense of shared purpose among network members. The 2010 workshops fostered understanding among the actors of constraints to the flow of information. The participatory nature of the workshops helped mobilize all the actors in the network and gain their buy-in for the SMS initiative. Participants in the 2011 workshops produced a graphic demonstration of striking differences in knowledge exchange in the very short time between baseline and endline. Discussions of Net-Map findings made it clear to participants that the impressive changes in the communications network for frontline health workers were due to the use of mobile phones and links made through the Hubs to the district supervisors and coordinators as sources of information and assistance.

Net-Map is an important tool for action research that produces robust data while enhancing knowledge sharing among research participants.

These workshops paved the way for sustaining the SMS initiative beyond the life of the project. The District Hospitals assigned individuals to monitor the Hubs, and they set aside funding for airtime. Ultimately, they were unable to maintain virus-free computers necessary to support the Hubs at the District Hospitals. Nevertheless, community health workers in both districts are continuing to use their mobile phones—at their own cost—to communicate with each other and with their supervisors.
